# Placode rotation in transitional lumbosacral lipomas: are there implications for origin and mechanism of deterioration?

**DOI:** 10.1007/s00381-018-3782-1

**Published:** 2018-03-29

**Authors:** Victoria Jones, Dominic Thompson

**Affiliations:** Great Ormond Street Institute of Child Health, 30 Guildford Street, London, WC1N 1EH UK

**Keywords:** Dysraphism, Lipomyelomeningocoele, Subtype, Neuro-orthopaedic syndrome, Magnetic resonance imaging

## Abstract

**Purpose:**

Rotation of the lipoma-neural placode has been noted in transitional lumbosacral lipomas. The purpose of this study was to confirm this rotation; that this rotation occurs with a preference to the left, and correlates with clinical symptoms. In addition, this study tests the hypothesis that this rotation occurs through local mechanical forces rather than intrinsic congenital malformation.

**Methods:**

Lipomas were classified as per the Chapman classification. Degree of rotation of the placode from the coronal plane was recorded along with the presence of herniation outside of the vertebral canal. Abnormalities on urodynamic testing were recorded, along with neuro-orthopaedic signs picked up on formal neuro-physiotherapy assessment.

**Results:**

Placode rotation occurs more frequently in the transitional group. Regardless of lipoma classification, rotation was much more common to the left. Furthermore, when lateralisation of symptoms was present, this strongly correlated with the direct of rotation. There was no difference in rotation of the placode whether it was within (lipomyelocoele) or without the vertebral canal (lipomyelomeningocoele).

**Conclusions:**

Placode rotation is a feature of transitional lumbosacral lipomas and may account for the increase in symptoms amongst this subgroup. Herniation of the placode outside the vertebral canal does not increase the risk of rotation suggesting a congenital cause for this finding rather than a purely mechanical explanation.

## Introduction

Lumbosacral lipomas (LSLs) are considered to be a form of closed neural tube defect. They account for the most frequent occurrence of closed spinal dysraphism (1 in 4000). Children present at a young age with cutaneous manifestations: a sacral swelling, focal hirsutism or pigmentation, often before neurological symptoms become apparent [5, 7, 20, 25]. As the name suggests, a mass of adipocytes are located at the caudal most aspect of the spinal cord. The mass of predominantly adipocytes is closely adherent to an abnormal caudal spinal cord; the fatty mass then extends through a defect within the dura, a defect in the vertebral lamina, and becomes continuous with the subcutaneous fat [9, 12, 20, 22]. The timing and extent of surgery remains controversial [16–18, 24], whilst the embryogenesis of this pathology also remains unproven with no animal model in existence [4].

Chapman has classified LSLs based on radiology and surgical anatomy into dorsal, caudal and transitional types according to the site of attachment of the lipoma relative to the conus medullaris [5]. In the dorsal subtype, the interface between lipoma and neural placode is above the conus; the roots of the cauda equina are separate to the lesion and the surgical anatomy tends to be more favourable. By comparison, in the caudal subtype, the tip of the conus becomes continuous with the lipoma and there is a variable association with the roots of the cauda equina. The transitional subtype is allied to the caudal type in that the conus is also involved, the interface between lipoma and neural placode extending for a variable distance from the conus along the dorsolateral aspect of the terminal spinal cord, there is invariable involvement and asymmetry of the roots of the cauda equina. Attempts have been made to correlate radiological findings and anatomical subtype with prognosis; however, although findings have been inconsistent most neurosurgeons would agree that the transitional subtype portends a more severe long-term prognosis, particularly in terms of sphincter continence [23].

In the transitional subtype, the attachment of the lipoma to the neural placode is rarely symmetrical; rather, this interface is typically rotated to one side [10, 20, 22]. This rotation results in some nerve roots emerging more dorsally and therefore having a longer course to their respective nerve root exit foramina whilst the contralateral nerve root are located deeper and are shorter being closer to their exit foramina. To what extent this rotation is congenital rather than a local mechanical response to growth of the spine and lipoma is unknown; however, the nerve roots on either side are frequently irregular in size, number and point of attachment to the conus suggesting a significant congenital component.

LSLs can be further classified as being associated with herniation of the meninges outside of the vertebral canal, often associated with co-herniation of the caudal spinal cord and neural placode, often referred to as a lipomyelomeningocoeles (LMM). Alternatively, no herniation of the meninges through the bony defect is often referred to as a lipomyelocoele (LM).

There has been some evidence that neural placode rotation occurs preferentially to one side more than the other. In addition, this asymmetry has also been documented in the location of cutaneous stigmata and manifestations of neuro-orthopaedic syndrome [20]; however, no correlation has yet been established between rotation, symptoms and prognosis. There is, however, an increasing literature on the early developmental origins of laterality and it is postulated that if there were significant tendencies to rotate to one side, this might shed some light on the pathogenesis of lumbosacral lipoma.

The aim of this study is to confirm the presence of placode rotation within LSLs, and in particular that this rotation occurs not only more commonly in the transitional subtype, but also occurs more commonly towards the left. Secondly, we propose that rotation puts mechanical stress on nerve roots and that a rotated neural placode is likely to be correlated with the presence of unilateral symptoms. Finally, we propose that this laterality and rotation can be due to either congenital effects or local mechanical influences. Since herniation of the cord out of the canal is likely to cause significant mechanical effects, one would expect to see a significant difference when herniation is present (lipomyelomeningocoeles) compared to when it is not present (lipomyelocoeles). Alternatively, no difference between lipomyelomeningocoeles and lipomyelocoeles would be more consistent with a primary congenital origin of the rotation.

## Methods

Cases of transitional and non-transitional LSLs were identified from a spinal lipoma database collected at the Great Ormond Street Hospital (GOSH) over a period of 15 years. Classification was confirmed, as per Chapman classification, by both radiological assessments of T1-weighted axial and sagittal images and at time of surgery. In the case where serial imaging was done over a period of years, all pre-operative images were reviewed. Imaging was further reviewed to identify the neural placode and lipoma with classification of the neural placode as being rotated to either right, left or no significant rotation. Operation notes were then reviewed to confirm radiological rotation.

**Fig. 1:**
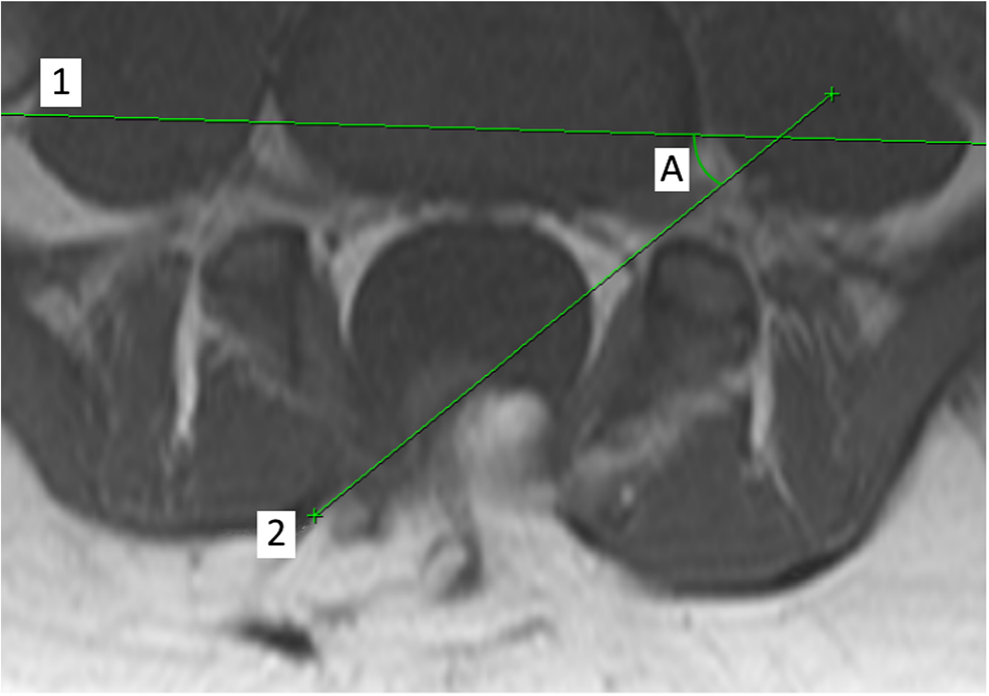
Measurement of rotation on T1 axial MR imaging with lipoma-neural placode rotated to the left. Lines that are drawn through coronal plane of vertebral body (1) and coronal plane of lipoma-neural placode at maximal rotation (2). Degree of rotation (A) measured as angle subtended by the plane of the placode (2) and horizontal plane (1)

Direction of rotation as recorded was taken to describe the position of the lipoma in respect to the spinal cord. A placode rotated to the right would be associated with lipoma tissue predominantly extending to the right of the spinal cord whilst nervous tissue remained to the left of the spinal canal. The right nerve roots would be orientated more ventrally and therefore deeper whilst the left nerve roots would be orientated more dorsally. Conversely, a lipoma said to be rotated to the left (Fig. 1) will be associated with a right-sided nerve root lying more superficial and presenting itself earlier at surgical dissection, whilst the left nerve root will lie deep and buried under the mass of the lipoma and the rotated neural placode.

T1-weighted axial and sagittal sections were reviewed to identify the point of the greatest rotation. Axial sections were then further reviewed with identification and marking of the midline. Care was taken to take into account possible rotation of the patient at time of imaging. This was achieved by determining the horizontal plane through the coronal axis of the vertebral body. A separate line was then drawn through the neural placode at the level of greatest rotation. The angle between this and the horizontal was then measured. No rotation was taken to be a neural placode orientated parallel to the horizontal plane. Results were subsequently divided into left rotation with the neural placode rotated anti-clockwise from the neutral position and right rotation with the neural placode rotated clockwise from the neutral position.

Any angle less than 20° was taken to be equivocal and not demonstrating any rotation. The thinking behind this decision was firstly to account for any potential error within the method described but secondly to account for the significance of rotation to surgical planning with rotation of less than 20° presenting no significance. Results were subsequently divided into 20–45°, 45–70° and 70–90°.

Clinical notes were then reviewed of the transitional LSLs to look for presence of urological symptoms from history and signs from formal urodynamic testing. Similarly, lateralised neurological symptoms such as pain were identified through the history and neuro-orthopaedic signs were documented from assessment by both a physiotherapist and a neurosurgeon.

Results are expressed as patient numbers followed by a percentage and confidence interval (expressed in brackets). Standard error was calculated to determine the 95% confidence interval, unless otherwise stated. Comparisons between test and control groups were performed by calculating the difference in percentage followed by the standard error of that difference. Results were taken to be significant when the range of the 95% confidence interval did not cross zero.

## Results

A total of 155 cases were reviewed using pre-operative magnetic resonance imaging and intra-operative surgical documentation. LSLs were classified as per Chapman classification.

A total of 52 cases of transitional lumbosacral spinal lipomas were identified and included in the final analysis. Twelve (23.1, 13.7–36.1%) cases were considered to show equivocal or no rotation (as mentioned above, this was taken to be less than a maximum of 20° rotation in either direction). Of the remaining 40 cases, 26 (65.0, 49.5–77.9%) showed rotation to the left between 20 and 90°. Conversely, 14 (35.0, 22.1–50.5%) cases showed rotation to the right between 20 and 90°.

Forty-six control patients with non-transitional LSLs were selected by random number generator and their pre-operative imaging reviewed. Thirty (65.2, 50.8–77.3%) demonstrated no or equivocal rotation, of the remaining 16, 11 (68.8, 44.4–85.8%) demonstrated rotation to the left and 5 (31.3, 14.2–55.6%) demonstrated rotation to the right.

**Fig. 2:**
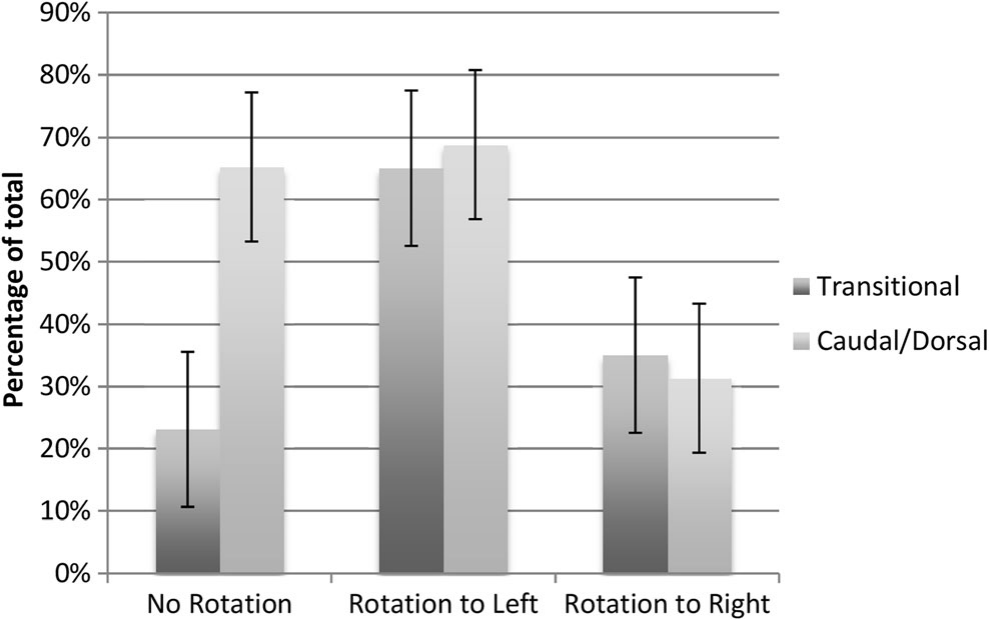
Comparison of incidence of direction of rotation between transitional and caudal/dorsal lipomas. Increased rotation is found in transitional lipomas; however, when rotation is present, the incidence of rotation to the left remains consistent between both groups

Relative risk of rotation was calculated between the transitional lipoma and non-transitional lipoma group to 2.21. Those children with transitional lipomas are 2.21 times more likely to have a rotated placode—an increase in 121%. Although rotation was much more common amongst the transitional lipomas, there was no significant difference between the directions of rotation between the two groups (Fig. 2).

**Fig. 3:**
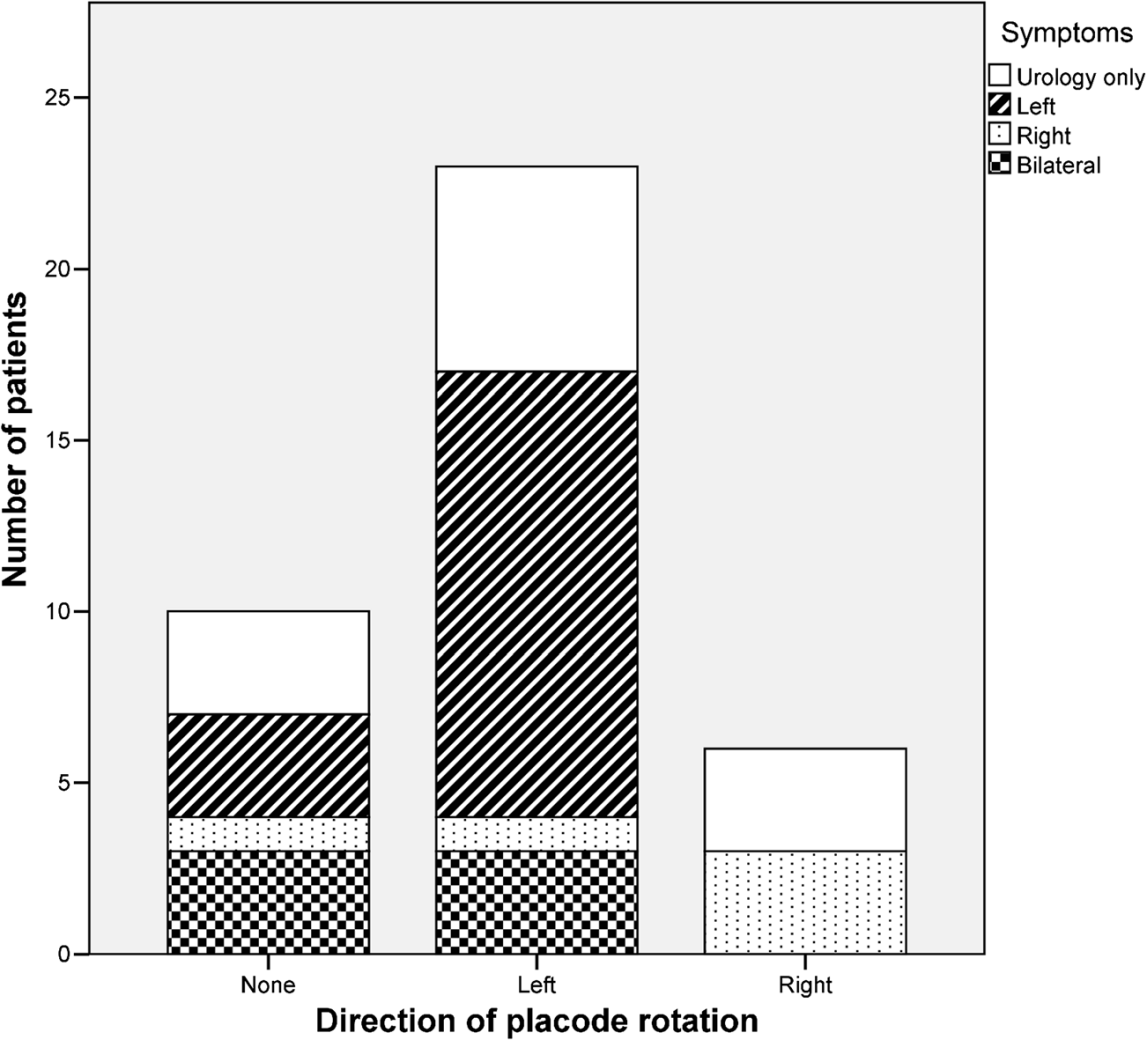
Symptoms experienced by patients with radiologically rotated transitional lipoma. Urological symptoms/abnormal findings on formal urodynamic assessment present with similar frequency in all groups. Side of symptoms associated with direction of rotation

Twelve (23%) patients were asymptomatic, a further 13 (25%) had only urological symptoms or abnormal findings on urodynamic assessment. A total of 27 (52%) patients had symptoms and/or signs in keeping with neuro-orthopaedic syndrome such as lower limb pain/altered sensation, muscle weakness and foot deformity. There was a clear correlation between direction of rotation of placode and unilateral symptoms (rotation to the left and left sided symptoms 76%, rotation to the right and right sided symptoms 100%) (Fig. 3).

Seventeen lipomyelomeningocoeles were identified accounting for 32% of cases and 35 lipomyelocoeles were identified accounting for 67% of cases. Further analysis was then performed to identify any difference in rotation between these two subgroups.

Of the 17 lipomyelomeningocoeles, 8 showed rotation to the left (57.1, 32.6–78.6%) and 6 showed rotation to the right (42.9, 21.4–67.4%), whilst 3 cases (17.6, 6.2–41.0%) demonstrated less than 20° rotation or were considered to show equivocal rotation. There was no significant statistical difference between the directions of rotation within this group.

**Fig. 4:**
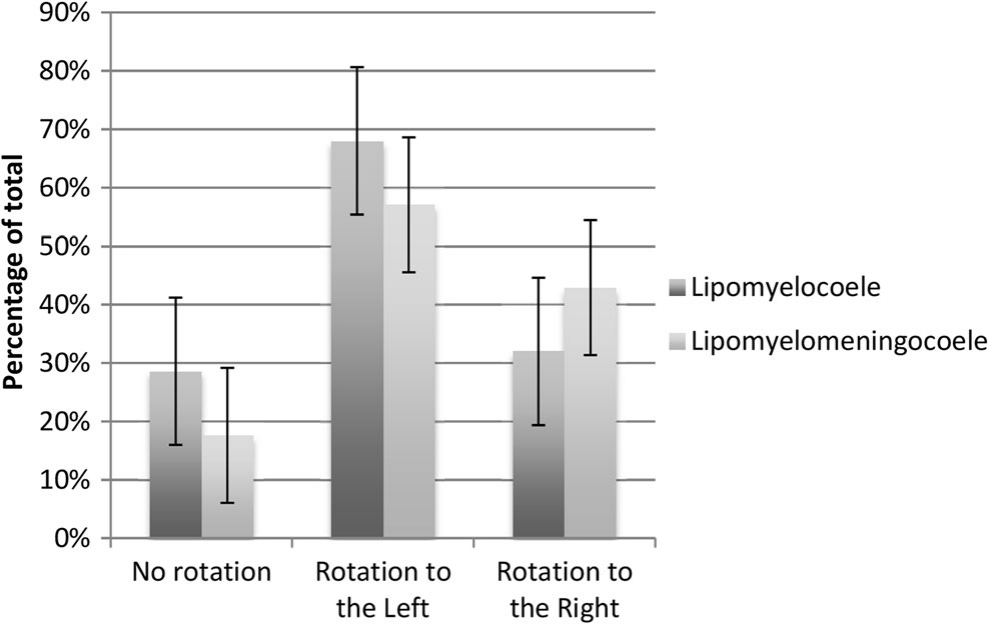
Comparison of incidence of direction of rotation between lipomyelocoeles and lipomyelomeningoceles. There is no significant difference between the two groups

Of the 35 lipomyelocoeles, 17 showed rotation to the left (68.0, 48.4–82.8%) and 8 showed rotation to the right (32.0, 17.2–51.6%), whilst 10 (28.6, 16.3–45.1%) demonstrated less than 20° rotation or were considered to show equivocal rotation. There was no significant statistical difference between the frequency and direction of rotation between the lipomyelocoele and lipomyelomeningocoele groups (Fig. 4).

To further test the hypothesis that placode herniation is associated with increased rotation, the degree of rotation was measured and recorded in groups: < 20°, 20–45°, 45–70° and 70–90°. None of the transitional LSLs demonstrated more than 90° rotation. When lipomyelomeningocoeles were rotated to the right, this was most frequently at 70–90° rather than lesser degrees of rotation. Lipomyelocoeles did not demonstrate this same preponderance for being maximally rotated to the right (Tables 1 and 2).

**Table 1:** Degree of rotation of neural placode

	L70–90	L45–70	L20–45	± 20	R20–45	R45–70	R70–90	Total
LM	6	4	7	10	3	4	1	35
LMM	3	4	1	3	1	1	4	17
TOTAL	9	8	8	12	4	5	5	52

*L* indicates rotation to left, *R* indicates rotation to right

**Table 2:** Degree of rotation of neural placode expressed as percentage of total cases by subtype

	L70–90	L45–70	L20–45	± 20	R20–45	R45–70	R70–90	Total
LM	17.6	11.8	20.6	26.5	8.8	11.8	2.9	100
LMM	17.6	23.5	5.9	17.6	5.9	5.9	23.5	100
TOTAL	17.6	15.7	15.7	23.5	7.8	9.8	9.8	100

*L* indicates rotation to the left, *R* indicates rotation to the right

## Discussion

Transitional lumbosacral lipomas are amongst the more difficult lipomas to treat surgically and are those associated with more significant long-term functional impairment. The majority of infants with lipomas are asymptomatic at initial presentation but over half will go onto deteriorate and manifest features of the neuro-orthopaedic syndrome. The pathogenesis of the neurological dysfunction in complex lipomas has been extensively debated; however, the relative contribution of congenital dysplasia of the terminal spinal cord versus mechanical tethering remains unknown. Hitherto, the lumbosacral lipomas have been considered as a single entity; however, this study of only transitional-type lumbosacral lipomas reveals significant anatomical heterogeneity in this group that might have implications for both our understanding of the cause of lipomas and the variable natural history.

The presence of nerve roots passing through the lipoma itself and the close adherence of the lipoma to nervous tissue at the neural placode requires meticulous dissection with the aid of neurophysiology to ensure the optimum outcome [16–19]. By reviewing our case series of transitional lipomas, we have demonstrated, for the first time, this variation in anatomy of this subtype of LSL.

We have demonstrated an association between transitional LSLs and rotation of the neural placode with rotation being 2.21 times more likely to occur in transitional LSLs. This raises the question as to why this is preferentially occurring in transitional LSLs as supposed to the other subtypes. We have tested the hypothesis that local mechanical factors may have a role by comparing rotation in the presence of placode herniation against the degree of rotation found with no herniation and have not found a significant difference. This highlights the possibility of an intrinsic congenital cause for the formation of transitional lipomas.

We have further demonstrated a correlation with direction of placode rotation and the presence of symptoms on the ventrally tilted side of the placode. It may be tempting to attribute this entirely to the mechanics of a rotated placode with one nerve root being considerably more stretched than the contralateral nerve root. However, pure rotation is likely to put stretch on both nerve roots. This, along with unilateral symptoms in non-rotated lipomas, supports the hypothesis that there is another intrinsic congenital process underlying the pathogenesis and progression of this pathology.

The pathogenesis of lumbosacral lipomas remains undetermined despite a range of theories [4, 7, 13, 15]. It is clear from the spina bifida defect associated with these lipomas that this is a congenital pathology with initiation of pathogenesis occurring prior to the completion of the formation of the caudal vertebrae. Neither a genetic nor an environmental cause has yet been found to cause LSLs, although some large scale genetic screens have suggested associations [3]. The presence of a degree of laterality demonstrated within this paper, with particular reference to transitional LSL, cannot explain the pathogenesis. However, it does raise interesting questions about the pathogenesis process: whether these lipomas form on one side of the embryo body axis, perhaps as a somatic mutation or through “premature disjunction” or whether local anatomy restricts growth of the lipoma such that it preferentially grows on the left more than the right, remains to be answered.

Normal development results in the asymmetry throughout systems of the body, perhaps most noticeably in the cardiovascular and gastrointestinal system. However, more subtle asymmetry is also present within the central nervous system. Regulation of this asymmetry is largely thought to be due to two mechanisms: firstly, the early expression of lefty2 and nodal on the left hand side of the body, and secondly, ongoing signals released by midline structures. Axis determination occurs early in the embryo with the node, primitive streak and even early endoderm all known to be involved in establishing the left-right body axis. Cilia within the node are thought to help develop a morphogen gradient with local leftwards laminar flow set-up by the nodal cilia. In addition, midline structures are also thought to act as a barrier to diffusion of these signals [11]. Early defects, such as in nodal, result in body wide defects—such as situs inversus. Notch has recently been found to have a role in establishing L-R asymmetry, of note, notch is also involved in the differentiation of adipocytes, smooth muscle, blood vessels and neural progenitors to glial cells [2, 6, 14, 21].

In LSLs, none of the surrounding non-spinal anatomy seems to be asymmetrical indicating an otherwise normal axis development. This suggests two hypotheses as to how L-R body patterning might be involved in LSL formation. Firstly, a germ-line mutation might be present which is susceptible to and only manifests in pathology in the presence of local signals. Alternatively, a somatic mutation might be acquired to the left or right, with left-sided mutations occurring more frequently due to the influences of local factors. A comparison can perhaps be made with Holt–Oram syndrome in which skeletal manifestations are more commonly seen on the left [8]. Although the transcription factor TBX5 is known to be mutated in inherited cases an explanation for this laterality remains absent [1].

The analysis within this paper is limited by the small number of cases of lumbosacral lipomas that are encountered, and this is particularly of importance when considering the further analysis of subtypes of transitional LSLs. However, for the first time, we demonstrate the frequency and degree of rotation of the neural placode within this pathology and a correlation with clinical symptoms. This ultimately might have implications towards understanding the embryogenesis of lumbosacral lipomas.
